# Efficacy of Dexmedetomidine With High-Flow Nasal Cannula Therapy in Children With Respiratory Distress: A Retrospective Case Series

**DOI:** 10.7759/cureus.99668

**Published:** 2025-12-19

**Authors:** Takaya Shimono, Kosei Yamashita, Sawa Seki, Toshiyuki Takagi, Megumi Okawa, Aiko Honda, Yuki Okada, Kazuki Kikuyama, Taro Watanabe, Katsumi Mizuno

**Affiliations:** 1 Department of Pediatrics, Showa University School of Medicine, Tokyo, JPN; 2 Department of Intensive Care Medicine, Showa University School of Medicine, Tokyo, JPN

**Keywords:** children, dexmedetomidine, high-flow nasal cannula, respirtory distress, side effects

## Abstract

Background

High-flow nasal cannulae (HFNC) are increasingly used in the management of respiratory distress in children. In pediatric patients, intubation should be considered if HFNC or noninvasive ventilation fails to improve respiratory status. Intubation is also indicated in the presence of upper airway obstruction or a sudden decline in consciousness. Dexmedetomidine (DEX), a sedative agent that can be administered without compromising spontaneous respiration, has recently gained attention. Research on DEX use under non-invasive ventilation (NIV) is increasing; however, data regarding the efficacy of combining HFNC with DEX are limited. We hypothesized that adding DEX to HFNC would reduce signs of respiratory distress in children and decrease the intubation rate.

Methods

This single-center, retrospective case series was conducted at Showa University Hospital. Patients admitted to the intensive care unit (ICU) between April 1, 2021, and August 31, 2023, with respiratory distress and managed with both HFNC and DEX were included. No comparator HFNC-alone group was available. The primary outcome was respiratory rate, assessed at 3 and 12 h after the initiation of DEX administration.

Results

Thirteen patients were included. The median age was nine (3-28) months, and respiratory syncytial virus was the cause in eight cases. The median respiratory rate upon ICU admission was 60 (56-64) breaths/min. At 3 and 12 h after treatment initiation, respiratory rates significantly decreased to 41 (31-52) and 40 (36-50) breaths/min, respectively (p<0.05).

Conclusions

Combining DEX with HFNC may reduce respiratory distress and help prevent the need for tracheal intubation in pediatric patients.

## Introduction

Bronchiolitis is the most common disease causing respiratory distress and respiratory failure in children [1,2], and respiratory syncytial virus (RSV) is the most common virus causing respiratory infection in infants [3]. Approximately 3-6 million children aged <5 years are hospitalized annually for RSV infections, and an estimated 100,000 deaths are reported worldwide [4]. In Japan, approximately 120,000-140,000 infants aged <2 years are diagnosed with RSV infection yearly, and approximately a quarter of them require hospitalization [5].

The management of respiratory diseases in children, including bronchiolitis, typically involves standard oxygen therapy (SOT). Since the 2000s, high-flow nasal cannula (HFNC) has been introduced for patients whose respiratory status is not stabilized with SOT [6]. HFNC is now increasingly used for respiratory management, along with nasal continuous positive airway pressure (CPAP) and bilevel positive airway pressure (BiPAP), as forms of non-invasive ventilation (NIV). A previous study comparing HFNC and SOT for infants with bronchiolitis reported that HFNC was more effective in providing increased respiratory support and reducing intensive care unit (ICU) stays [7]. However, even with HFNC, the respiratory status of some patients may deteriorate, necessitating intubation and mechanical ventilation.

Sedation during NIV has garnered increasing interest in recent years [8,9]. Dexmedetomidine (DEX) is an α2-selective adrenergic receptor agonist that exerts minimal direct effects on the respiratory center and tends to preserve airway reflexes, distinguishing it from other sedatives. Accordingly, DEX may be preferable during spontaneous breathing [10,11]. Because appropriate sedation can facilitate ventilation [12], combining DEX with HFNC may be more effective for respiratory management than HFNC alone. DEX may reduce respiratory effort and, when used with HFNC, may help decrease tachypnea and overall respiratory effort. Prior studies in pediatric patients have reported that DEX can help prevent perioperative respiratory adverse events, including cough, hypoxemia, and bronchospasm [13,14].

However, no prior studies have specifically examined whether the use of HFNC combined with DEX for respiratory distress in children improves respiratory stability and reduces the need for ventilatory support. Therefore, this study aimed to investigate whether combining HFNC with DEX reduces signs of respiratory distress and avoids tracheal intubation in pediatric patients presenting with respiratory compromise.

We hypothesized that the target sedative concentration of DEX in pediatric intensive care is typically reached within 3 h, maintained at 12 h, and associated with a reduction in respiratory rate at both 3 and 12 h [15]. The null hypothesis was that the respiratory rate between pre-DEX baseline and post-DEX measurements would not differ significantly.

## Materials and methods

Study design and population

This was a retrospective case-series study. Patients aged ≤15 years admitted between April 1, 2021, and August 31, 2023, were included. All patients were admitted to Showa University Hospital for respiratory distress, experienced deterioration under HFNC management, were admitted to the ICU, and received DEX. All procedures were approved by the Ethics Committee of Showa University School of Medicine (2024-289-B; approved February 20, 2025).

Selection criteria

Patients who were unable to maintain adequate respiratory status at HFNC flow rates of ≥2 L/kg/min and required ICU admission were included. Deterioration of respiratory status was defined as either a requirement for a fraction of inspired oxygen (FiO₂) >0.60 to maintain oxygen saturation ≥92% or worsening respiratory effort [16]. Worsening respiratory effort was defined as an increase in retractions, tachypnea, or the presence of shoulder use, nasal flaring, or seesaw breathing.

To minimize selection bias and clearly define the cohort, we applied the following exclusion criteria. First, patients whose respiratory support was switched to non-HFNC modalities (CPAP or BiPAP) before or after ICU admission were excluded. Second, cases with missing heart rate, respiratory rate, or respiratory effort data at 3 and 12 h after DEX initiation were excluded. Only cases with complete data at both time points were included in the analysis. Respiratory rate was routinely documented by the attending physician in the electronic medical record. Blood pressure was measured noninvasively approximately four to eight times daily. Respiratory distress was defined as use of accessory muscles with subcostal and intercostal retractions, tachypnea for age, and grunting [16].

Outcome definition

The primary outcome was the change in respiratory rate at 3 h after initiation of DEX administration. Secondary outcomes included respiratory rate at 12 h, presence of respiratory effort at 3 and 12 h, need for tracheal intubation, and safety outcomes. Safety was assessed by evaluating the presence of bradycardia, hypotension, and desaturation. The DEX infusion was reduced by 0.1 μg/kg/h when bradycardia exceeded the safety threshold. Bradycardia was defined as a heart rate below the 10th percentile for age [17]. Hypotension was defined as values below the fifth percentile for age, according to Pediatric Advanced Life Support criteria. Hypertension was defined as blood pressure above the 90th percentile for age [18]. Hypopnea was defined as a respiratory rate below the fifth percentile for age. Desaturation was defined as hypopnea with SpO_2_ desaturation less than 90% and/or the need to titrate NIV; events meeting these criteria were considered clinically significant. This definition aligns with that previously reported [19].

Additionally, differences in background characteristics between intubated and non-intubated patients at 12 h were evaluated. In some cases, sedatives such as triclofos sodium and chloral hydrate were co-administered with DEX. Comparisons were made to determine whether the addition of other sedatives was associated with a higher incidence of bradycardia.

Infusion protocol

The maintenance dose of DEX ranged from 0.2 to 0.7 μg/kg/h, with an initial loading dose administered at 6 μg/kg/h over the first 10 min [20]. The decision to administer a loading dose was made by the attending physician. If bradycardia, hypotension, or hypertension occurred, the DEX dosage was reduced or discontinued at the physician’s discretion. Additional sedatives, such as chloral hydrate or triclofos sodium, were administered if the patient’s respiratory condition worsened. Additional sedatives, such as chloral hydrate or triclofos sodium, were administered if the patient's respiratory condition worsened. Chloral hydrate was used at a dose of 250 mg for patients younger than three years and 500 mg for patients three years and older. Triclofos sodium was used at 40-70 mg/kg/dose as deemed appropriate by the attending physician.

Statistical analysis

Continuous variables are presented as medians with interquartile ranges. The association between the use of sedatives other than DEX and the incidence of bradycardia before and at 3 and 12 h after sedative administration was assessed using the Mann-Whitney U test. Statistical significance was set at p<0.05. Analyses were conducted using JMP software (version 17.0; SAS Institute Inc., Cary, NC, USA).

## Results

**Figure 1:**
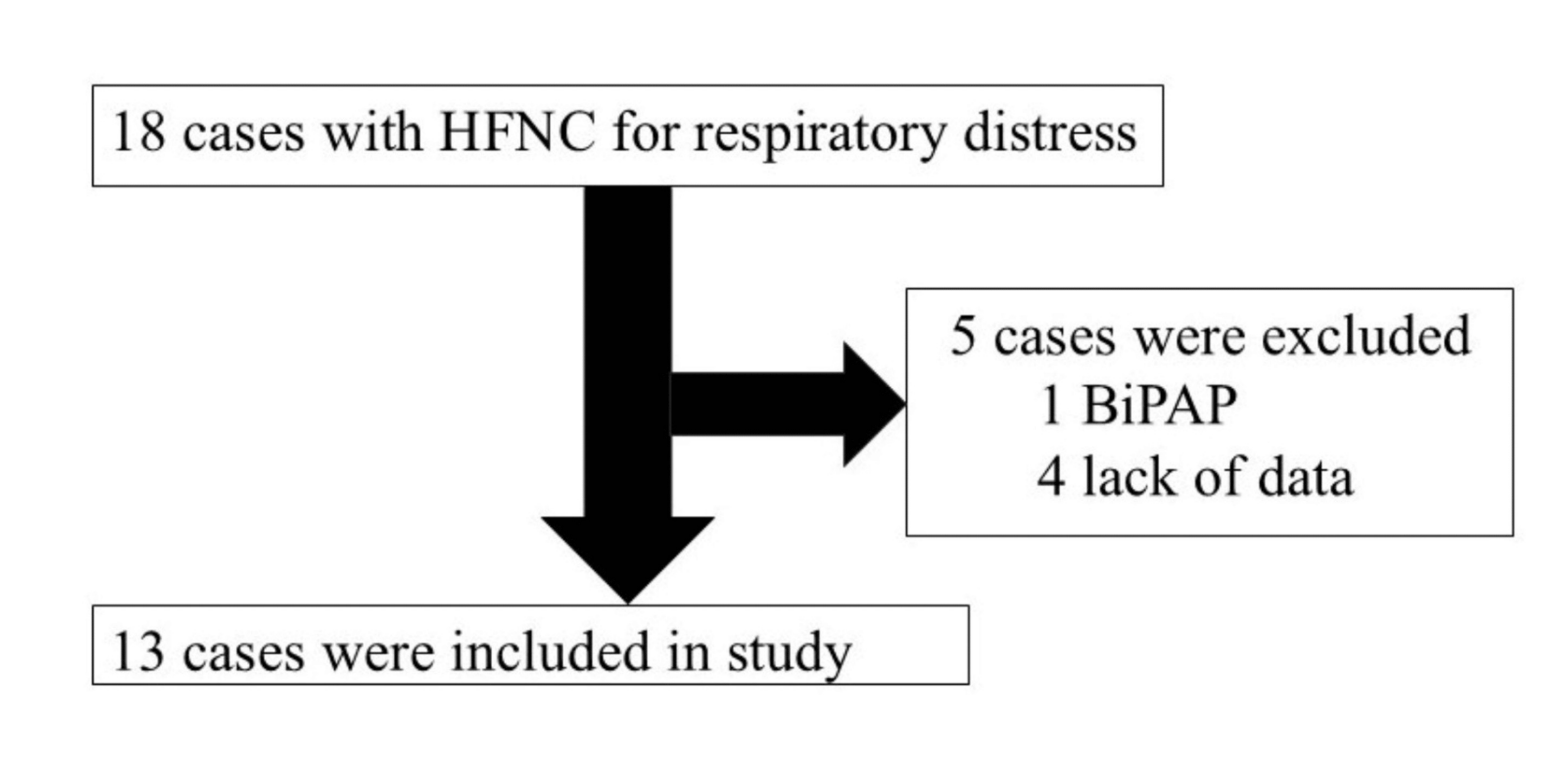
Flowchart of the study population BiPAP: Bilevel positive airway pressure.

Thirteen patients were included (Figure 1).

Nine (69%) were boys, and two (15%) had underlying chronic lung disease (CLD) and congenital heart disease (Table 1).

**Table 1:** Demographic and clinical characteristics of the patients at baseline Variables are expressed median (IQR) or number (%). pCO_2_: Partial pressure of carbon dioxide; HFNC: high-flow nasal cannula; DEX: dexmedetomidine.

	N=13
Age (months)	9 (3-28)
Weight (kg)	8.5 (4.0-10.0)
Sex (male, %)	9 (69)
Chronic lung disease (%)	2 (15)
Congenital heart disease (%)	2 (15)
Respiratory syncytial virus positive (%)	8 (62)
Human metapneumovirus positive (%)	1 (8)
Parainfluenza virus positive (%)	1 (8)
Heart rate (beat/min)	162 (142-180)
Respiratory rate (breath/min)	60 (56-64)
pH	7.34 (7.30-7.41)
pCO_2_ (mmHg)	44〔38-56〕
Use of other sedative drugs	9 (69)
HFNC initial flow rate(L/min)	15〔15-25〕
DEX maximum dose(μg/kg/h)	0.4〔0.35-0.6〕

**Figure 2:**
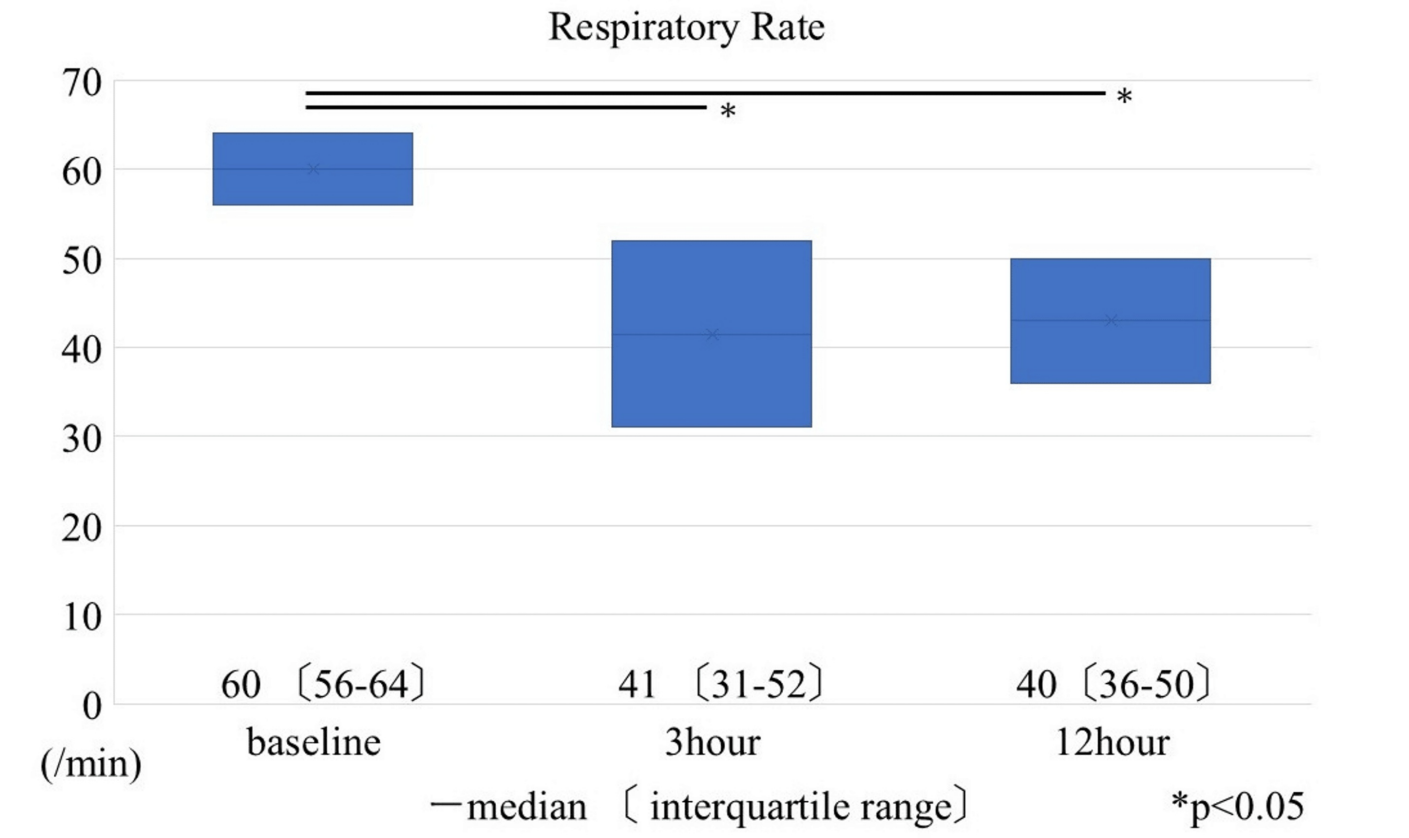
Respiratory rate at 3 and 12 h after the initiation of dexmedetomidine administration

The primary outcome, respiratory rate, is shown in (Figure 2).

**Figure 3:**
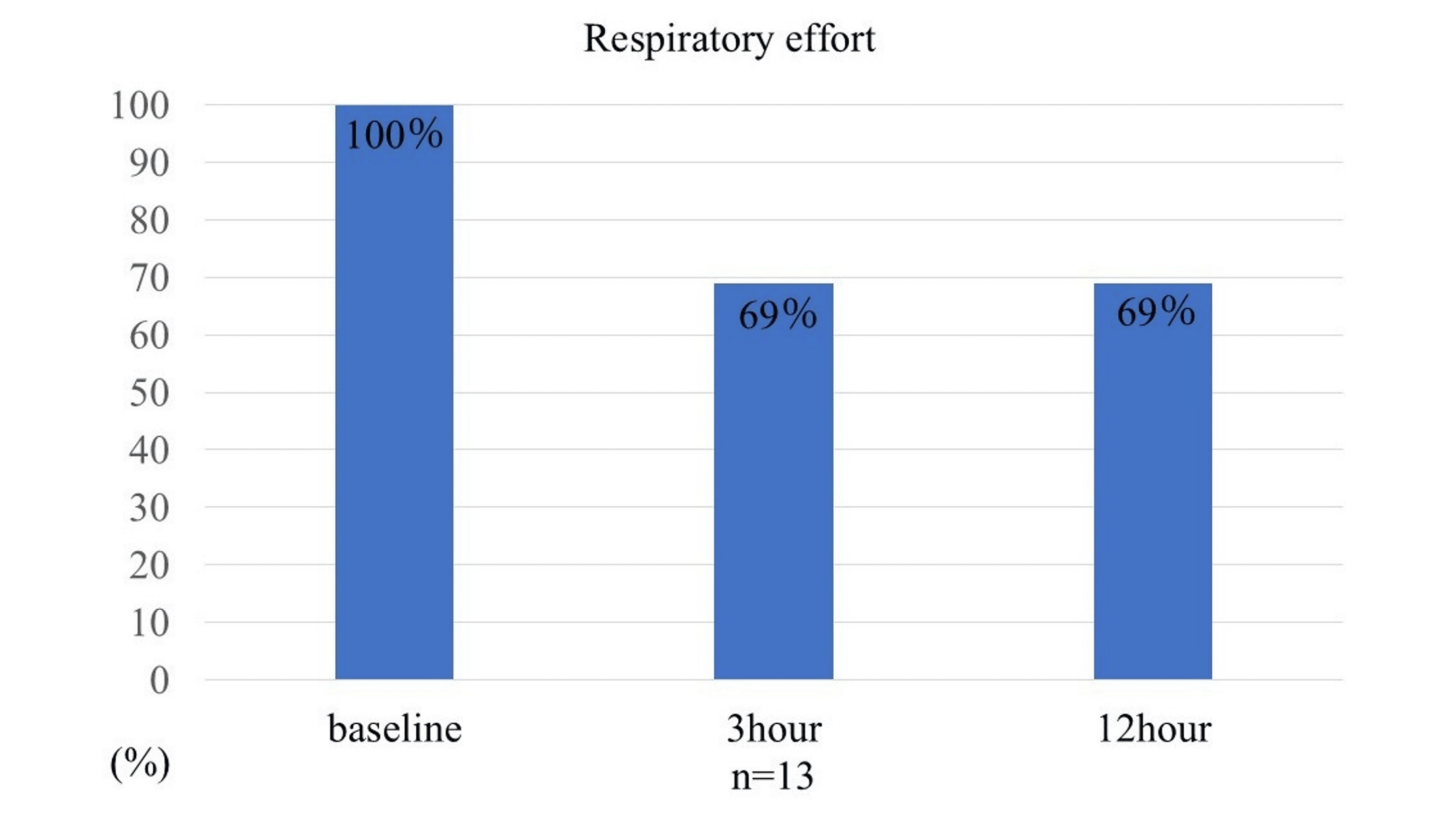
Respiratory effort at 3 and 12 h after the initiation of dexmedetomidine administration

Respiratory rate decreased significantly from baseline. The median respiratory rate was 60 (56-64) breaths/min at initiation and decreased to 41 (31-52) breaths/min at 3 h, corresponding to a 26% to 18% reduction from baseline (95% confidence interval (CI)). At 12 h, the median respiratory rate was 40 (36-50) breaths/min, representing a 27% to 19% reduction (95% CI). Both reductions were statistically significant (p<0.01 vs baseline). Respiratory effort was observed in all patients prior to sedative administration and resolved in four patients (31%) at both 3 and 12 h (Figure 3).

None of the patients required intubation at 3 h, while three required intubation by 12 h. Data on ICU admissions for both intubated and non-intubated patients are presented in (Table 2).

**Table 2:** Intubated and non-intubated cases with residual respiratory effort after 12 hours Variables are expressed median (IQR) or number (%). pCO_2_: Partial pressure of carbon dioxide.

	intubation (n=3)	no intubation (n=10)	P value
Age (months)	28 (7-48)	6 (2-12)	0.46
Weight (kg)	8.6 (5.8-10.0)	7.6 (4.8-10.5)	0.45
Sex (male, %)	1 (33)	8 (80)	0.13
Chronic lung disease (%)	2 (67)	0	0.01
Congenital heart disease (%)	1 (33)	1 (10)	0.32
Respiratory syncytial virus positive (%)	2 (67)	6 (60)	0.84
Human metapneumovirus positive (%)	1 (33)	0	0.06
Parainfluenza virus positive (%)	0	1 (10)	0.57
Heart rate (beats/min)	131 (127-207)	161 (145-180)	0.45
Respiratory rate (breath/min)	61 (58-67)	59 (50-63)	0.42
pH	7.40 (7.30-7.43)	7.33 (7.28-7.41)	0.21
pCO_^2^_ (mmHg)	38.4 (34.5-55.6)	45.0 (41.1-59.1)	0.45

Children with CLD were more likely to require intubation; however, no other significant differences were identified between the two groups. We performed a subgroup analysis comparing intubation rates between patients with CLD and those without among the 13 patients who received HFNC with DEX. Intubation was significantly more frequent in patients with CLD (100% (2/2)) than in those without CLD (9.1% (1/11)) (Fisher’s exact test, p=0.039). This finding limits the generalizability of the observed reduction in intubation to populations with a high CLD burden. While the therapy appears effective for acute respiratory distress without chronic underlying disease, caution is warranted when applying these results to patients with established structural lung changes or reduced pulmonary reserve, such as those with CLD. Further studies are needed to clarify optimal respiratory support strategies for patients with impaired pulmonary mechanics. Nine patients were administered sedatives other than DEX. The proportion of bradycardia in patients treated with the combination and DEX was 6/9 (67%) and 3/4 (75%), respectively. No statistically significant difference was observed in the rate of bradycardia between the groups (p=0.53). Bradycardia resolved in all cases after reducing or discontinuing DEX. One patient (8%) developed hypertension, which resolved after dose reduction. No patient developed hypotension or desaturation.

## Discussion

The use of DEX with HFNC in children showing signs of respiratory distress appears to contribute to improved respiratory status. More than half of the patients may have required intubation under conventional respiratory management, which was avoided through the use of DEX. Thus, DEX administered under HFNC may enhance respiratory status and reduce the need for intubation in pediatric patients. The pharmacological properties of DEX helped alleviate tachypnea and labored breathing while preserving spontaneous respiration. Effective sedative blood concentrations can be achieved at any pediatric age when DEX is administered at a rate of 0.7 µg/kg/h following an initial loading dose of 1 µg/kg over 10 min [21]. As shown in Figure 3, respiratory effort had resolved in four patients by 12 h after DEX initiation, consistent with adequate sedation. Of the nine patients with persistent respiratory effort, three required intubation. Although not all patients with persistent effort progressed to intubation, the risk remains clinically relevant. CLD appeared to be a risk factor for intubation, and prior reports note higher intubation rates in RSV infection when CLD is present, supporting our observation [22]. While conventional management calls for intubation when HFNC fails to improve respiratory status, we observed that several patients avoided intubation, suggesting that DEX used with HFNC may help reduce the need for intubation.

To the best of our knowledge, only three previous studies have reported the use of DEX under NIV in children and its associated adverse effects [19,23,24]. The primary adverse events associated with DEX are dose-dependent hemodynamic effects, including bradycardia, hypotension, and hypertension [10,19,23]. Compared with prior reports, we observed a higher incidence of bradycardia and a lower incidence of hypertension. Interpretation is limited by the small sample size, and these estimates may change as more cases accrue. This discrepancy may reflect the lower maintenance DEX dose used in our cohort relative to prior studies (0.2-1.5 μg/kg/h) [19,23,24]. A comparison with previous reports is shown in Table 3.

**Table 3:** Pediatric case of DEX under NIV management ^a^Only Eidman et al. report maximum and minimum values; others report interquartile ranges. ^b^Heart rate and respiratory rate are both defined in the fifth percentile. ^c^Bradycardia is defined in the 10th percentile. ^d^Heart rate and blood pressure were not defined. ^e^The percentages in parentheses conform to the definition of bradycardia established by Shutes et al. The definition of non-invasive ventilation (NIV) included the use of continuous positive airway pressure (CPAP), bilevel positive airway pressure (BiPAP), or high-flow nasal cannula.

Author	Number of cases	DEX dose(μg/kg/h)^a^	Bradycardia	High blood pressure	Low blood pressure	Desaturation
Venkatraman et al.(2017) [19]^b^	202	0.4-0.8	13%	No report	20%	5%
Shutes et al.(2018) [23]^c^	382	0.6-1.2	28%	33%	2%	No report
Eidman et al.(2022) [24]^d^	68	0.2-1.2	15%	No report	12%	No report
This report	13	0.2-0.6	23%(69%^e^)	8%	0%	0%

At low doses, DEX exerts sympatholytic effects leading to bradycardia and hypotension, while higher doses may cause peripheral vasoconstriction and hypertension [11]. In our cohort, bradycardia was common. Although statistical significance was not achieved, we cannot exclude the possibility that other sedative agents used concomitantly contributed to the bradycardia observed. In all cases of bradycardia, reducing the DEX infusion by 0.1 μg/kg/h resolved the event.

Multiple adverse events associated with DEX have been reported, including those noted in this study. For instance, a prior study reported cardiopulmonary resuscitation for apnea caused by bradycardia-induced cardiac arrest during DEX administration [19]. Other documented effects - bradycardia, hypotension, and hypertension - typically improved with dose reduction or discontinuation. These findings suggest that DEX may be safe when administered with careful monitoring of vital signs. While DEX has minimal effects on respiration compared to other sedatives, clinicians should remain vigilant regarding its circulatory impact. Its minimal respiratory depression makes it a potentially safe sedative option under HFNC for both children and adults experiencing respiratory distress.

All patients met clinical criteria for intubation due to persistent respiratory effort despite HFNC use [16]. In adults with respiratory failure, NIV with DEX has been shown to reduce intubation rates [25]. However, given the retrospective design and small sample size of our study, further investigation, particularly of intubation outcomes, is warranted. Although our post hoc subgroup analysis showed a higher intubation rate among patients with CLD, the very small sample limits the generalizability of this finding; large studies are needed to confirm this association.

Our study has some limitations. First, the primary limitation is the small sample size, which reduces statistical power and limits the generalizability of the findings. As the number of cases increases, interpretation of results regarding specific side effects may change. We therefore plan to extend the study period and reanalyze the data to further investigate the effectiveness of DEX. Second, because this was a retrospective study, we could not standardize the timing, dosage, or depth of sedation across patients, which may have introduced variability in sedation levels. We also lacked objective measures of respiratory distress and were unable to apply validated tools such as the Silverman-Anderson score; consequently, we relied on subjectively documented clinical observations of respiratory effort. Third, a definitive conclusion regarding the therapeutic effect of DEX cannot be drawn because the study lacked a control group receiving HFNC monotherapy. Fourth, the sedative effect may have been influenced by confounding, as several participants concurrently received other sedatives, making it difficult to isolate the independent effect of DEX. Finally, baseline characteristics (e.g., underlying conditions) varied, and sedative dosing was not fully standardized; these factors constrain reproducibility and warrant caution.

Although all patients were successfully extubated and discharged, we could not assess long-term outcomes, such as the duration of respiratory support or recurrence of respiratory distress. A large, prospective study is needed to evaluate these longer-term outcomes.

## Conclusions

In conclusion, HFNC combined with DEX may be effective for respiratory management in children. Interpretation should be cautious given the small sample size, absence of a control group, and potential confounding. If effective, this approach may improve respiratory status, reducing tachypnea and respiratory effort, and may also decrease the need for invasive procedures such as endotracheal intubation. Large, prospective randomized controlled trials that control for confounders are needed to confirm efficacy and better assess causality.
